# A New CDK2 Inhibitor with 3-Hydrazonoindolin-2-One Scaffold Endowed with Anti-Breast Cancer Activity: Design, Synthesis, Biological Evaluation, and In Silico Insights

**DOI:** 10.3390/molecules26020412

**Published:** 2021-01-14

**Authors:** Mohammad M. Al-Sanea, Ahmad J. Obaidullah, Mohamed E. Shaker, Garri Chilingaryan, Mohammed M. Alanazi, Nawaf A. Alsaif, Hamad M. Alkahtani, Sultan A. Alsubaie, Mohamed A. Abdelgawad

**Affiliations:** 1 Department of Pharmaceutical Chemistry, College of Pharmacy, Jouf University, Sakaka 72341, Aljouf Province, Saudi Arabia; mohamedabdelwahab976@yahoo.com; 2 Department of Pharmaceutical Chemistry, College of Pharmacy, King Saud University, Riyadh 11451, Saudi Arabia; mmalanazi@ksu.edu.sa (M.M.A.); nalsaif@ksu.edu.sa (N.A.A.); ahamad@ksu.edu.sa (H.M.A.); 439106132@student.ksu.edu.sa (S.A.A.); 3 Department of Pharmacology, College of Pharmacy, Jouf University, Sakaka, Aljouf 72341, Saudi Arabia; mshaker2222@yahoo.com; 4 Department of Pharmacology and Toxicology, Faculty of Pharmacy, Mansoura University, Mansoura 35516, Egypt; 5 Institute of Biomedicine and Pharmacy, Russian-Armenian University, Yerevan 0051, Armenia; garri.chilingaryan@rau.am; 6 Department of Pharmaceutical Organic Chemistry, Beni-Suef University, Beni-Suef 62514, Egypt

**Keywords:** synthesis, CDK2, 3-hydrazonoindolin-2-one, MCF-7 breast cancer cell line, cytotoxicity, apoptosis

## Abstract

Background: Cyclin-dependent kinases (CDKs) regulate mammalian cell cycle progression and RNA transcription. Based on the structural analysis of previously reported CDK2 inhibitors, a new compound with 3-hydrazonoindolin-2-one scaffold (**HI 5**) was well designed, synthesized, and biologically evaluated as a promising anti-breast cancer hit compound. Methods: The potential anti-cancerous effect of **HI 5** was evaluated using cytotoxicity assay, flow cytometric analysis of apoptosis and cell cycle distribution, ELISA immunoassay, in vitro CDK2/cyclin A2 activity, and molecular operating environment (MOE) virtual docking studies. Results: The results revealed that **HI 5** exhibits pronounced CDK2 inhibitory activity and cytotoxicity in human breast cancer MCF-7 cell line. The cytotoxicity of **HI 5** was found to be intrinsically mediated apoptosis, which in turn, is associated with low Bcl-2 expression and high activation of caspase 3 and p53. Besides, **HI 5** blocked the proliferation of the MCF-7 cell line and arrested the cell cycle at the G2/M phase. The docking studies did not confirm which one of geometric isomers (*syn* and *anti*) is responsible for binding affinity and intrinsic activity of **HI 5**. However, the molecular dynamic studies have confirmed that the *syn*-isomer has more favorable binding interaction and thus is responsible for CDK2 inhibitory activity. Discussion: These findings displayed a substantial basis of synthesizing further derivatives based on the 3-hydrazonoindolin-2-one scaffold for favorable targeting of breast cancer.

## 1. Introduction

Cyclin-dependent kinases’ (CDKs) family members are protein kinases that have an important role in regulating the sequential stages of the cell cycle [1]. In particular, CDK2 has a key role in cell cycle progression from the late G1-phase to the S-phase alongside initiating DNA repair [2]. Otherwise, CDK2-dependent cell cycle regulation is critical for proliferation, proliferation, growth, and maintenance of different cancer tumors [3,4]. For instance, breast cancer falls under the most prevalent and lethal cancer types affecting women worldwide [5]. Several studies have demonstrated that inhibition of CDK2 could induce breast cancer cell apoptosis without damage to normal cells [6,7]. Because of their substantial role in cell cycle regulation, CDKs have become attractive therapeutic targets for limiting cancers in the breast and those occurring in the pancreas, gastrointestinal tract, kidney, and other organs [1,4,8,9,10].

A vast number of studies has been performed to discover and develop new selective CDK2 inhibitors for cancer therapy with minimal off-target toxicity. For instance, the flavone alkaloid alvocidib (flavopiridol) was the first multi-CDK inhibitor (types 1, 2, 4, and 9) to be studied in clinical trials but had limited efficacy and high toxicity [11,12]. Dinaciclib with a pyrazolo [1,5-α]pyrimidin-7-amine core structure is a potent and selective CDK2 activity with an IC_50_ value of 1 nM under phase III clinical trial [13,14]. Milciclib is another CDK2 kinase inhibitor with a 4,5-dihydro-1*H*-pyrazolo[4,3-h]quinazolin-8-amine scaffold that showed selectivity toward CDK2 (IC_50_ = 45 nM) at least three times more than other CDKs 1, 2, 4, 5, and 7 [15,16]. Besides, the small molecule roscovitine (seliciclib) inhibited CDKs 2 and 5 with suspending the proliferation of rapidly growing mammalian cancer cell lines with an IC_50_ value of 16 μM for CDK2 and induced growth arrest in G1-phase and accumulation in G2-phase [17,18,19]. The 3-substituted indolinone compound SU9516 is another inhibitor of CDK2 that preferably binds to the ATP binding site of CDK2 with an IC₅₀ value of 22 nM than other CDKs (40 nM for CDK1 and 200 nM for CDK4) [20]. Lastly, NU6300 is the first irreversible CDK2 modulator that can bind covalently to the cysteine residue in the ATP binding site [21,22,23]. Different indole and indole isosteres-based compounds were shown to potently inhibit cyclin-dependent kinase 2 (CDK2) activity as part of a G1 cell cycle arrest of human breast cancer cells [23,24,25,26,27,28,29]. A variety of oxindole scaffold-based compounds was synthesized as potential modulators for CDKs. Sunitinib is an FDA approved anticancer agent with an oxindole core structure, and it showed multikinase inhibitory activity towards CDK2, VEGF1-3, PDGFRb, RET, FLT3, and CSF-1R [30,31].

**Figure 1:**
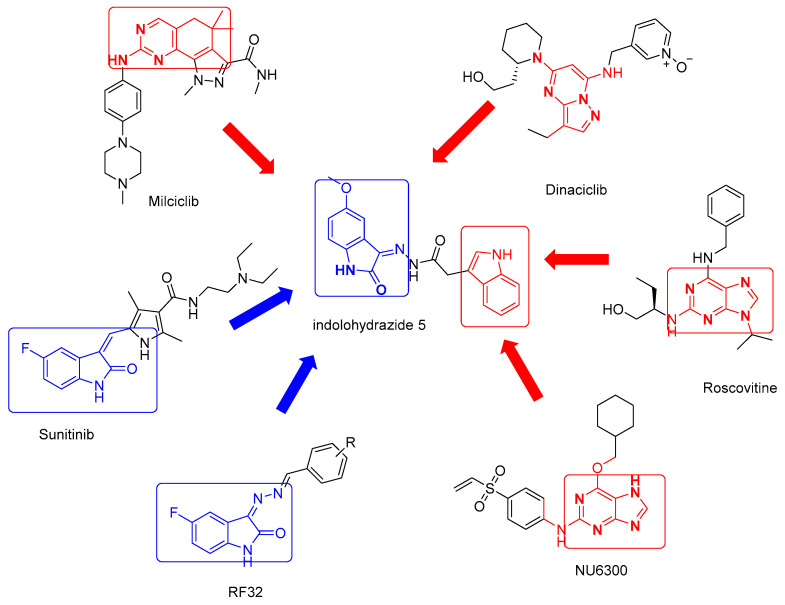
The structures of some potent CDK2 inhibitors.

Based on the structure analysis, most of the inhibitors mentioned above share an amino-substituted heterocyclic scaffold, which plays a crucial role in developing binding affinity on CDK2. Despite the recent improvements in the discovery of CDK2 inhibitors, some restrictions that obstruct their applications at a clinical level, such as the resistance to the drug, lack of specificity, and efficiency. Therefore, dire needs to develop small molecule inhibitors that target CDK2 with potential and specificity to overcome resistance among cancer cell populations. Stirred by these findings and in connection with our current experimental work, it is thought beneficial to extend our investigations to examine a new 3-hydrazonoindolin-2-one scaffold-based compound (**HI 5**) as potential anti-breast cancer and pro-apoptotic agent. Utilizing the virtual docking, the design here is based on attaching two pharmacologically interested hydrophobic moieties (indole and isatin) via acetohydrazide linker (Figure 1). In this article, **HI 5** was designed, synthesized, and biologically evaluated as a potential anticancer agent. The target compound was evaluated for its cytotoxicity toward two human breast cancer cell lines MCF-7 and MDA-MB-231 and the doxorubicin-resistant ovarian cell line NCI-ADR utilizing SRB assay. Moreover, the growth inhibition mechanism in relation to cell cycle regulation and apoptosis induction in breast MCF-7 cells cancer cells through the DNA flow cytometry and Annexin V-FITC/PI assays were executed. Furthermore, inhibitory actions against CDK2 kinase and the anti-apoptotic protein Bcl-2 were implemented. Lastly, virtual docking simulations were performed to explore the binding modes of the titled compound within the CDK2 active site. The experimental results showed that the **HI 5** is a novel CDK2 inhibitor with promising antiproliferative activity against human breast MCF-7 and resistant ovarian NCI-ADR cell lines. Additionally, **HI 5** was able to block the cell cycle at G2/M and induce marked apoptosis in MCF-7 cells.

## 2. Results

### 2.1. Chemistry

**Scheme 1:**
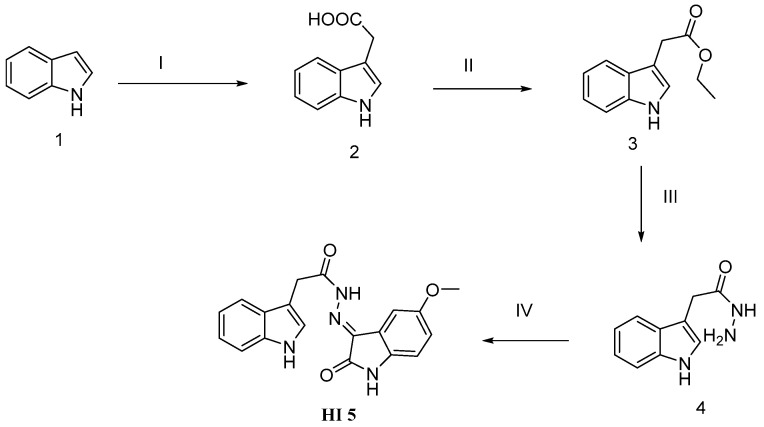
Schematic representation for **HI 5** synthesis: *Reagent and conditions:* (I) indole, glycolic acid; KOH, HCl, H_2_O; (II) ethanol, H^+^, reflux for 10 h; (III) hydrazine hydrate, ethanol, reflux for 2 h; (IV) acetic acid, 5-methoxyisatin, reflux for 4 h.

The new target indolohydrazid 5 (**HI 5**) was prepared through a four-steps reaction: the first step is coupling dehydration of glycolic acid with indole **1** to give indol-3yl-acetic acid **2**, which is esterified with ethanol in acidic medium to afford indole acetic acid ester **3**. The ester compound **3** was reacted with hydrazine hydrate to afford hydrazide **4**. 5-methoxyindole 2,3-dione condensed in anhydrous condition with **4** to produce the target **HI 5** (Scheme 1) using previously described methods for reported compounds (**2**–**4**) [32,33].

The aliphatic NMR peaks, whether ^13^C-NMR or ^1^H-NMR, confirm the postulated structure of **HI 5**. ^1^H-NMR peaks appeared at ẟ 3.72–3.76 (m, 3H, OCH_3_), 3.88 (s, 1H, CH_2_), 4.20 (s, 1H, CH_2_) are representing the aliphatic protons. Additionally, the ^13^C-NMR peaks, which appeared at ẟ 28.17, 32.36, 55.56, 55.66, confirmed the presence of aliphatic carbons.

### 2.2. Biological Screening

#### 2.2.1. CDK2 Inhibitory Activity

**Figure 2:**
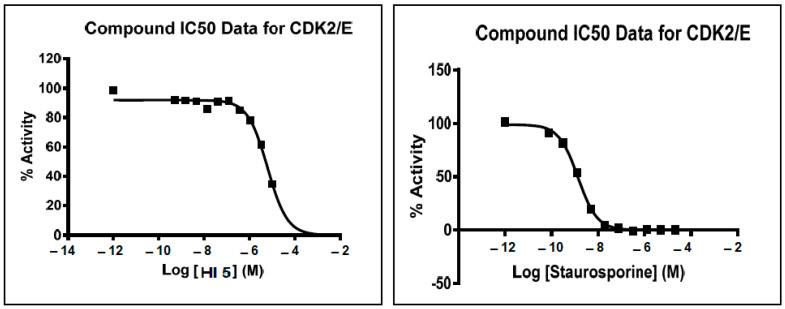
Effects of different concentrations of **HI 5** and staurosporine on CDK2/E enzyme activity.

Among the investigated protein kinases, **HI 5** showed moderate potency against CDK2 with single-digit micromolar EC_50_ value (6.32 µM) as shown in (Figure 2).

**HI 5** showed high growth inhibitory activity CDK2/A2 (49%) and moderate (27%) growth inhibitory effect against c-Met at a concentration of 10 µM (Table 1). Hence, **HI 5** exhibited certain selectivity toward CDK2/A2.

#### 2.2.2. Annexin V-FITC Apoptosis Assay

**Figure 3:**
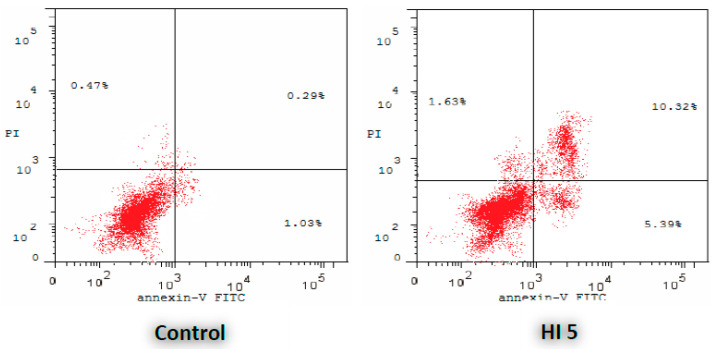
Effect of **HI 5** on the percentage of annexin V-FITC-positive staining in MCF-7 cells. The four quadrants identified as: **LL**, viable; **LR**, early apoptotic; **UR**, late apoptotic; **UL**, necrotic.

The data showed that cytotoxicity of **HI 5** is mainly apoptosis as indicated by an increase in the percentage of cells in lower (early apoptotic) and upper (late apoptotic) right quadrants (Figure 3 and Table 2).

#### 2.2.3. Antiproliferative Activities toward Breast Cancer Cell Lines

The antiproliferative effect of the titled compound has shown four times more potency than Dox in inhibiting the proliferation of the breast cancer cell line MCF-7, while it was found to be equipotent with Dox in inhibiting the proliferation of the breast cancer cell line MDA-MB-231 (Table 3). **HI 5** has also shown antiproliferative potency on the ovarian cancer cell line NCI-ADR (Table 3).

#### 2.2.4. The Effect on the Apoptotic and Anti-Apoptotic Marker Levels

Interestingly, **HI 5** lowered the cellular expression of Bcl-2 alongside raising Bax, leading to a more than a 10-fold increase in the Bax/Bcl-2 ratio compared to the untreated MCF-7 control cells. Interestingly, HI 5-treated cells have shown 10-fold higher expression of active Caspase 3 and 16 fold higher expression of p53 than untreated cells. Thus, the markers of mitochondrial damage and apoptosis are elevated in cancer cells by **HI 5** pretreatment (Table 4).

#### 2.2.5. Cell Cycle Assay

**HI 5** treatment caused accumulation of MCF-7 cells in the G2/M phase of the cell cycle, compared to the untreated control cells (Table 5). Thus, **HI 5** affected the cell cycle distribution of MCF-7 cells.

### 2.3. In Silico Studies

#### 2.3.1. Virtual Docking of **HI 5**

**Figure 4:**
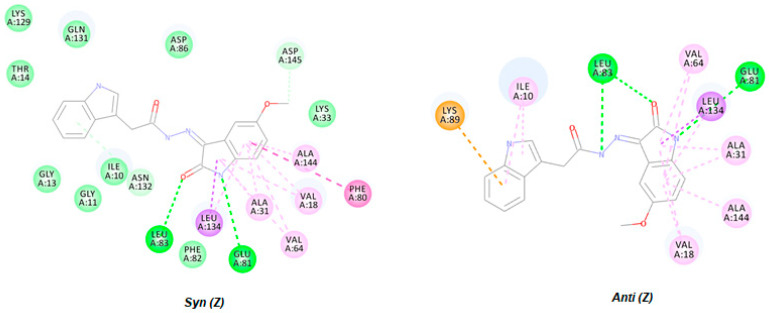
Two-dimensional representation of docking poses of **HI 5** geometric isomers (*E* and *Z*).

To compare the two geometric isomers of **HI 5**, the two isomers were subjected to virtual docking using MOE suite (Molecular Operating Environment (MOE), Chemical Computing Group Inc.: Montreal, QC, Canada, 2016. The ATP binding site of CDK2 is positioned deep at the joint of the C and N domains, including the pivotal catalytic amino acids with high sensitivity and kinase hinge amino acids (Phe82, Glu81, and Leu83). Docking simulation of both **HI 5** isomers (*E* and *Z*) showed different binding orientations inside the CDK2 binding pocket (Figure 4).

#### 2.3.2. Molecular Dynamics Simulations

**Figure 5:**
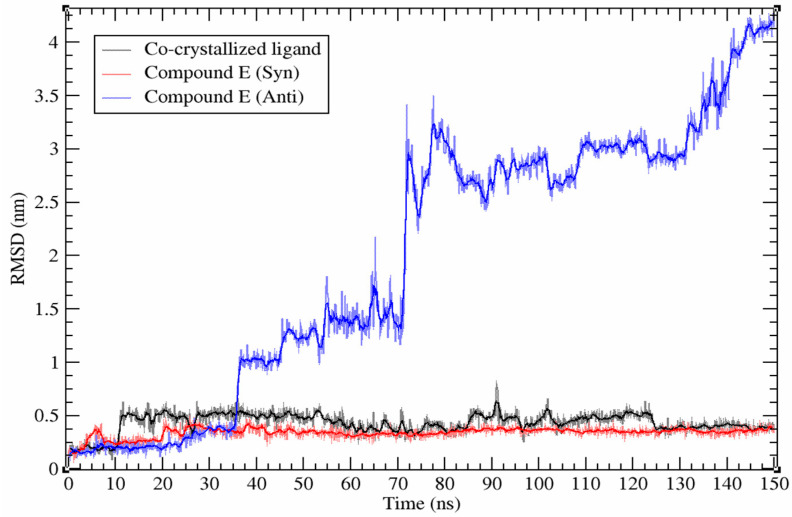
Root mean square deviation (RMSD) plot of **HI 5** (*syn* and *anti*-isomers) and co-crystallized ligand during simulations.

Simulations showed that syn conformation of the **HI 5** interacts with the CDK2 ATP binding site similar to the reference ligand fashion, while anti-isomer does not interact with it. The ligand’s dynamic stability was studied by calculating the root mean square deviation (RMSD) of the heavy atoms of the investigated compounds (Figure 5).

The co-crystallized ligand binds to the site with the average binding energy of −106.7 kJ/mol. *Syn*-isomer of the **HI 5** interacts with the ATP binding site with an average binding energy of −91.6 kJ/mol. Van der Waals, electrostatic, polar solvation, solvent accessible surface (SASA), and binding energies for the complex structures of **HI 5** isomers and CDK2 were calculated using the G_MMPBSA approach for the last 10 ns of the simulations and presented in Table 6.

**Figure 6:**
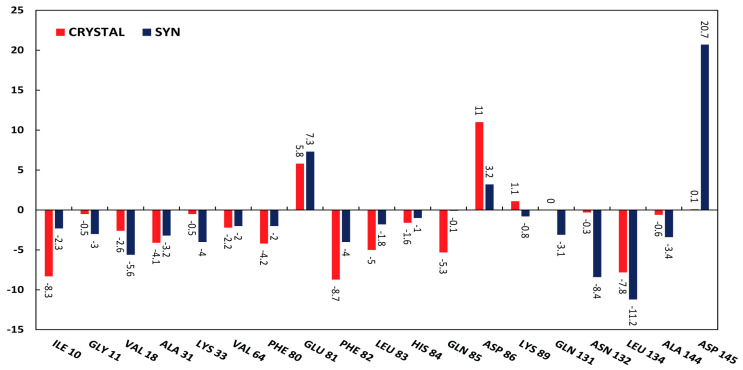
Comparison of interacting amino acid residues of the ATP binding site of CDK2 (PDB ID 1FVT) with **HI 5** *syn*-isomer (*Syn*) and co-crystallized ligand (Crystal).

To distinguish the key amino acid residues that play an essential role in binding, we calculated the energetic contributions of all amino acid residues to the ligand binding. Comparison and differences in the values of total energies of the ATP binding site’s amino acid residues with **HI 5** *syn*-isomer and co-crystallized ligand are presented in Figure 6.

**Figure 7:**
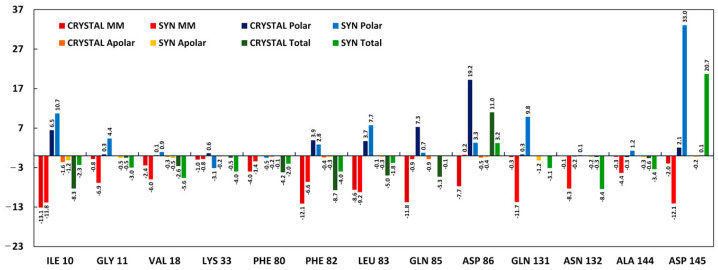
Energetic components’ values of the selected amino acid residues with **HI 5** *syn*-isomer (*Syn*) and co-crystallized ligand (Crystal).

Thirteen amino acids with a difference in energy values more than ~2 times were carefully compared to gain insights into four energetic components (ΔE*_MM_*, ΔG*_polar_*, ΔG*_apolar_*, and ΔG*_total_*). Figure 7 depicts the contribution of these four energetic components to the overall binding affinity.

## 3. Discussion

### 3.1. Chemistry

^1^H-NMR and ^13^C-NMR data were effectively used to elucidate the chemical structures of **HI 5**. The shielded ^1^H-NMR peaks at ẟ 3.72, 3.76, 3.88, and 4.20 represent the aliphatic protons OCH_3_ and CH_2_. The three NH groups are represented by six peaks in ^1^H-NMR at δ range of 10.89 to 13.01 ppm. ^13^C-NMR spectrum showed four aliphatic carbons at ẟ 28.17, 32.36, 55.56, 55.66, and the total 38 peaks confirm the presence of the two geometric isomers. The NMR spectra of **HI 5** showed characteristic double peaks in equal percentage, which indicated that the two isomers (*Syn*/*Anti*) were presented in an equal percentage [34,35].

### 3.2. Biological Screening

#### 3.2.1. CDK2 Inhibitory Activity

Protein kinases constitute a major class of enzymes that have importance in cell division and signaling [36]. Pharmacological inhibitors of certain protein kinase subtypes such as serine/threonine and tyrosine kinases represent the targeted cancer therapy class members. Accordingly, we determined whether cytotoxicity of **HI 5** is related to inhibition of certain types of serine/threonine and/or tyrosine kinases. Of the investigated protein kinases, **HI 5** showed marked inhibition of the activity of the serine/threonine kinase CDK2 acting on cyclin E with an IC_50_ of 6.32µM as shown in (Figure 2). Besides, **HI 5** exhibited high inhibitory activity (49%) against CDK2 acting on cyclin A and moderate inhibitory effect against the tyrosine kinase c-Met at a concentration of 10 µM (Table 1).

#### 3.2.2. Annexin V-FITC Apoptosis Assay

Induction of apoptosis is a well-known therapeutic strategy for mediating tumor remission. The ongoing research on chemotherapeutics works on developing novel small molecules that augment the extrinsic and intrinsic pathways of apoptosis. These apoptotic pathways are commonly impaired by the cancer cells’ resistance mechanisms to maintain their progression and evasion from the host immune surveillance. To test whether **HI 5** has potential cytotoxicity, flow cytometric analysis was performed using annexin V-FITC and PI stainings to detect apoptosis and necrosis of MCF-7 cells, respectively (Figure 3 and Table 2).

#### 3.2.3. Antiproliferative Activities toward Breast Cancer Cell Lines

Next, the antiproliferative potency of **HI 5** was compared with the classical chemotherapeutic agent doxorubicin (Dox) by determining the IC_50_ on breast MCF-7 and MDA-MB-231 cancer cell lines (Table 3). Besides, the antiproliferative effect of **HI 5** was further confirmed on resistant ovarian cancer cell line NCI-ADR (Table 3).

#### 3.2.4. The Effect on the Apoptotic and Anti-Apoptotic Marker Levels

The extrinsic pathway is mediated by exogenous stimulation of death receptors by their ligands, while the intrinsic apoptotic cell death pathway is initiated by endogenous signals arising from DNA damage and oxidative stress. These signals induce the mitochondrial permeability transition pore opening by inactivating the anti-apoptotic proteins such as Bcl-2. Thus, it is not surprising that cancer cells dampen these damaging signals by overexpressing Bcl-2. The overexpression of Bcl-2 also acts as an inhibitor for apoptotic proteins such as Bax to mediate the mitochondrial permeability transition pore opening. Therefore, the influence of **HI 5** on Bcl-2 and Bax expression levels in MCF-7 cancer cells was quantified to ascertain whether **HI 5** induces cytotoxicity via the mitochondrial permeability transition pore opening (Table 4). The data indicated that **HI 5** induced the mitochondrial permeability transition pore opening as evidenced by the higher Bax/Bcl-2 ratio, compared to control cells.

Following the mitochondrial permeability transition pore opening, cytochrome C is released from the mitochondria and activates caspase 9 via the apoptosome assembly. Subsequently, the activated caspase-9 cleaves and activates other executioner Caspases, such as caspase-3 and caspase-7, followed by DNA damage and apoptosis. Accordingly, **HI 5**′s effect on active caspase 3 and p53 expressions in MCF-7 cancer cells was evaluated as indicators of DNA damage and cell death (Table 4). Based on these results, **HI 5**-induced cytotoxicity is mediated by the mitochondrial intrinsic pathway of apoptosis.

#### 3.2.5. Cell Cycle Assay

The cell division cycle comprises G1 phase of cellular growth and preparation for DNA synthesis, S phase of DNA synthesis, G2 phase of preparation for mitosis, and M phase of mitosis. We investigated the effect of **HI 5** on the cell cycle. According to the results, we can determine whether **HI 5** is a cell cycle-specific agent that acts selectively on cancer cells when they are traversing, or not. (Table 5). **HI 5** treatment raised the percentage of MCF-7 cells in the G2/M phase of the cell cycle in comparison to the control cells (36% vs. 14%). Thus, **HI 5** seems to impair the phases of DNA replication and mitosis of cancer cells, leading to arrest of division.

### 3.3. In Silico Studies

#### 3.3.1. Virtual Docking of **HI 5**

The molecular docking simulations for **HI 5** in its two geometric isomers (*E* and *Z*) showed three important hydrogen bond interactions with Leu83, Glu81, and Leu83 via acetohydrazide NH and 2-oxoindolin N and 2-oxoindolin O, respectively, which represent key interactions with kinase active domain (Figure 4). Besides, the docking poses of **HI 5** showed that 2-oxoindolin moiety made anchoring hydrophobic interactions mixed with VDW interactions with Val18 and Val64 and Ala144 and Ala31 from both sides, as revealed in Figure 4.

#### 3.3.2. Molecular Dynamics Simulations

Results of molecular dynamics simulation of **HI 5** *syn*-isomer at the ATP binding site of the CDK2 showed highly stable (RMSD < 0.2 nm) binding affinity with the maintenance of initial binding pose during 150 ns simulation. However, the *anti*-isomer leaves the ATP binding site after ~36 ns then keeps localized close to binding site until ~70 ns followed by complete left until the end of the simulation (Figure 5). According to the performed molecular dynamics simulation results, the **HI 5** *anti*-isomer does not interact properly with the ATP binding site of the CDK2.

**HI 5***syn*-isomer and reference ligand interact with the ATP binding site of CDK2 with the close average binding energy (Table 6). Total interaction energies of the following amino acids: Ala31, Val64, Glu81, His84, Lys89, and Leu134 are similar for the two compounds. Glu81 and Asp86 have relatively high positive values of total binding energy with both compounds, while Asp145 has high total positive binding energy only with **HI 5***syn*-isomer. Gln85 interacts only with co-crystallized ligand with high negative binding energy. Asn132, in contrast to Gln85, interacts only with *syn*-isomer of the **HI 5**. Ile10, Phe80, Phe82, and Leu83 interact with co-crystallized ligand with relatively higher binding affinity than *syn*-isomer. At the same time, Gly11, Val18, Lys33, Gln131, Leu134, and Ala144 interact with higher binding affinity with the *syn*-isomer of **HI 5**.

Asp145 interacts with *syn*-isomer of the **HI 5** through relatively strong VdW and electrostatic energy (−12.1 kJ/mol), but overwhelmingly, high polar energy (33 kJ/mol) overall results in the repelling effect between **HI 5** *syn*-isomer and Asp145, while co-crystallized ligand does not interact with this residue. High polar energies in cases of Asp86 and Glu81 also result in repelling effects.

Both VdW and electrostatic energies control most of the analyzed interactions between amino acid residues of the ATP binding site of CDK2 and studied compounds. The similar binding affinity of the **HI 5** *syn* and co-crystallized ligand and the difference in the binding site’s interacting amino acid residues pattern characterize *syn*-isomer of the **HI 5**, a potential competitive inhibitor of the CDK2. The obtained results have valuable information that can be used for future design and modifications of the **HI 5**′s *syn*-isomer, in particular, to neutralize high polar energy values of the mentioned binding site’s amino acid residues.

## 4. Materials and Methods

### 4.1. Chemistry

Compound **1** was commercially available, and it was obtained from Sigma Aldrich Company, St. Louis, MO, USA. Compounds **2**–**4** were chemically synthesized according to the reported methods [37].

Synthesis of (*syn*/*anti*) 1*H*-indol-3-yl)-acetic acid (5-methoxy-2-oxo-1,2-dihydro-indol-3-ylidine)-hydrazide (HI 5). A mixture of (1*H*-indol-3-yl)-acetic acid hydrazide (0.01 mol, 1.89 gm) and 5-methoxy-1*H*-indole 2,3-dione (0.01 mol, 1.77 gm) was dissolved in absolute ethanol (30 mL) containing glacial acetic acid (0.01 mL) [38,39,40]. The reaction mixture was heated under reflux for 6h, and the reaction progress was followed by TLC. The reaction mixture was cooled, and the formed precipitate was filtered and crystallized from ethanol with yield of 62%. Yellow solid, m.p.: 232.5−234 °C, ^1^H-NMR (DMSO-*d*_6_, 400 MHz) δ ppm; 3.72–3.76(m, 3H, OCH_3_), 3.88(s, 1H, CH_2_), 4.20(s, 1H, CH_2_), 6.78–6.891(m, 2H, ArH), 6.96–7.07(m, 2.5 H, ArH), 7.18 (s,0.5H, ArH), 7.30–7.36(m, 2H, ArH), 7.51–7.60(m, 1H, ArH), 10.89–11.08(m, 2H, 2NH), 12.54(s, 0.5H, NH), 13.01(s, 0.5H, NH). ^13^C-NMR (DMSO-*d*_6_, 100 MHz) δ ppm: 28.17, 32.36, 55.56, 55.66, 105.49, 105.60, 105.74, 106.35, 107.05, 111.44, 111.50, 111.55, 111.89, 117.38, 117.82, 118.25, 118.46, 118.60, 118.65, 120.63, 121.04, 121.20, 121.32, 124.39, 124.83, 126.99, 127.10, 127.27, 134.02, 135.95, 136.20, 136.32, 137.02, 137.20, 155.34, 162.60, 168.42, 173.40. MS *m*/*z* [%]: 348.7 [M^+^, 13.44], 190.5 [M^+^, 100]; elemental analysis for C_19_H_16_N_4_O_3_; Calc. C; 65.51, H; 4.63, N; 16.08, found C; 65.22, H; 4.69, N; 15.98.

### 4.2. Antiproliferative Activities Toward Breast and Resistant Ovarian Cancer Cell Lines

Breast (MCF-7 and MDA-MB-231) and doxorubicin-resistant ovarian (NCI-ADR) cancer cell lines were obtained from Nawah Scientific Inc., (Mokatam, Cairo, Egypt). In Dulbecco’s Modified Eagle’s media, cells were maintained and supplemented with 100 mg/mL of streptomycin, 100 units/mL of penicillin, and 10% heat-inactivated fetal bovine serum in humidified, 5% (*v*/*v*) CO_2_ atmosphere at 37 °C.

### 4.3. Cytotoxicity Assay

Cell viability was assessed by SRB assay according to the reported procedure. Aliquots of 100 μL cell suspension (5 × 10^3^ cells) were taken in 96-well plates and incubated for 24 h. Cells were treated with another aliquot of 100 μL media containing the vehicle DMSO or the HI5 at various concentrations. After 72 h of drug exposure, cells were fixed by replacing media with 150 μL of 10% TCA and incubated at 4 °C for 1 h. The cell wells were then washed five times with distilled water, followed by the addition of 70 μL of SRB solution (0.4% *w*/*v*) and incubation in the dark at room temperature for 10 min. Plates were washed three times with 1% acetic acid and allowed to air-dry overnight. Then, 150 μL of TRIS (10 mM) was added to dissolve the protein-bound SRB stain; the absorbance was measured at 540 nm.

### 4.4. Flow Cytometric Analysis of Apoptosis and Cell Cycle Distribution

MCF-7 cells were treated by **HI 5** at IC_50_ concentration or the vehicle DMSO for 24 h. After that, the cells were collected for flow cytometry detection of exposure of internal membrane phosphatidylserine occurring during apoptosis using staining with Annexin-V-FITC (BD Pharmingen, San Diego, CA, USA) and propidium iodide (PI, Sigma-Aldrich, St. Louis, MO, USA) using FACS Calibur and CellQuest software (Becton Dickinson, Totowa, NJ, USA). Flow cytometry was also performed for MCF-7 cells after PBS wash, ethanol fixation, ribonuclease, and PI additions when treated or not treated by **HI 5** at IC_50_ concentration for 24 h to analyze the distribution of cell cycle phases as previously described [38] (See supplementary file).

### 4.5. ELISA Immunoassay

The concentrations of Bax, Bcl-2, cleaved caspase-3, p53, and phosphorylated c-Met were determined using ELISA kits (Abcam, Boston, MA, USA). In brief, MCF-7 cells were treated by **HI 5** at IC_50_ concentration or the vehicle DMSO for 24 h. After that, the cells were rinsed with PBS and lysed in RIPA lysis buffer at 4 °C for 30 min, followed by centrifugation at 10,000× *g* for 20 min. Supernatants were then collected and stored at −80 °C for later protein determination using Bradford’s protein assay. Then, equal protein concentrations of cell lysates were incubated in pre-coated ELISA kits with primary antibodies specific to Bax, Bcl-2, and cleaved caspase-3. After one hour, the appropriate secondary biotin-linked antibody was added, followed by streptavidin-HRP and TMB/H_2_O_2_. A stop solution was finally added to the plate wells when the developed color was optimal, followed by measuring the optical absorbance at 450 nm by a microplate reader (BMG LABTECH-FLUO star Omega, Ortenberg, Germany) (See supplementary file).

### 4.6. In Vitro CDK2/Cyclin A2 and c-Met Activity

The CDK2/cyclin A2 and c-Met protein kinase assays were performed using Kinase-Glo Plus luminescence kinase assay kit (Promega, Madison, WI, USA), as illustrated earlier [19,24,41]. In brief, the kinase activity was assessed by measuring the unreacted ATP remaining in solution after the kinase reaction. The percentage inhibition of the **HI 5** against CDK2/cyclin A2 and c-Met protein kinases were compared to that of staurosporine at a single concentration of 10 µM (See supplementary file).

### 4.7. In Silico Studies

#### 4.7.1. Virtual Docking of **HI 5**

All molecular docking studies were performed using molecular operating environment (MOE) software. The crystal structure of CDK2 was downloaded from the RCSB Protein Data Bank (PDB ID: 1FVT). The protein crystal structure was then prepared, and the docking protocol was validated and performed according to the reported methodologies [28,42].

#### 4.7.2. Molecular DYNAMICS simulations

Missing atoms of the Leu25, Arg36, and missing amino acid residues (Leu37, Asp38, Thr39, Glu40, Thr41, Glu42, Gly43, Val154, Pro155, Val156, Arg157, Thr158, Tyr159, Thr160, His161, Glu162) of the CDK2 structure (PDB ID:1FVT) were modeled using CHARMM-GUI [43]. Three molecular dynamics simulations: *syn*/*anti*-isomers of the **HI 5** and co-crystallized ligand with CDK2, were performed, every 150 ns. Docking binding poses of *syn* and *anti*-isomers of **HI 5** were used as starting points for the corresponding simulations; co-crystallized ligand (106) of the 1FVT was used as a reference compound [44]. Molecular dynamics simulations were performed using GROMACS 2019.25 molecular dynamics package. AMBER99SB-ILDN force field was used for simulations [45,46]. As the force field does not contain parameters for the ligand, all ligands were parameterized using ANTECHAMBER to generate consistent parameters with the General Amber Force Field (GAFF) [41]. The AM1-BCC method was used to assign charges. ACPYPE was used for conversion of ligands topologies to format compatible with GROMACS [45,46,47]. All simulations were performed in an explicit water environment, using the TIP3P model. Complexes were solvated in a dodecahedron box system and were neutralized with Na^+^ and Cl^−^ ions. Steepest descent was used for depreciation, and Fmax was set of no greater than 1000 kJ mol^−1^ nm^−1^. Systems were equilibrated using NVT and NPT ensembles for 200 ps duration each. The temperature was sustained at 300 K using the V-rescale algorithm [48]. For the regulation of systems pressure, the Parrinello–Rahman barostat was used. The linear constraint solver (LINCS) algorithm was used for the bond’s length constraints [49]. The particle mesh Ewald (PME) method was used for long-range electrostatics calculations. For all simulations, time step was set to 2 fs. Van der Waals’s cut-off distance was set to 1 nm. Binding-free energies were calculated using the G_MMPBSA program [50,51], with the MM-PBSA method adapted for GROMACS. The binding energy consists of three energetic terms, potential energy in a vacuum, polar-solvation energy, and non-polar solvation energy. The SASA model was used to calculate the non-polar solvation energy. For the calculations of all interaction energies, the last ten ns (every frame with 100 ps interval) of the trajectories were used.

## 5. Conclusions

In conclusion, we have successfully designed and synthesized a novel molecule with 3-hydrazonoindolin-2-one scaffold (**HI 5)**. This compound has shown marked cytotoxic activity on certain human breast cancer cell lines through intrinsic apoptosis. The antiproliferative effects against MCF7 cell line showed an IC_50_ of 1.15 µM. The cell cycle assay showed growth arrest of MCF7 cell lines at G2/M phase. Compound **HI 5** showed selectivity toward CDK2/A2 with high (49%) growth inhibitory activity against CDK2/A2. The molecular docking studies of **HI 5** in the CDK2 active site disclosed that the indole and isatine groups could be exchanged with possible other bioisosteric equivalents with different Es, π, and σ values. Moreover, the *syn*- and *anti*-isomers of **HI 5** showed almost the same binding types, strength, and energy score. However, the molecular dynamic simulations confirm the responsibility of *syn*-isomer (rather than the *anti*-isomer) for the CDK2 antagonistic activity of **HI 5**. Further development of 3-hydrazonoindolin-2-one scaffold will be applied to synthesize a new series of bioisosteric analogs with more potent and selective CDK2 inhibitory activities.

## Figures and Tables

**Table 1:** Inhibitory effect of **HI 5** on CDK2/A2 and c-Met kinases activity at a single dose of 10 µM.

**Compound**	**% Enzyme Inhibitory Activity**	
	CDK2/A2	c-Met
**HI 5**	49	27
Staurosporine	100	100

**Table 2:** Distribution of apoptotic cells in the AnnexinV-FITC/PI dual staining assay in MCF-7 cells after treatment with **HI 5**.

Comp.	Early Apoptosis (Lower Right (LR) %)	Late Apoptosis (Upper Right (UR) %)	Total (LR % + UR %)
**HI 5**	5.39	10.32	15.71
Control	1.03	0.29	1.32

**Table 3:** In vitro antiproliferative activity of **HI 5** against breast (MCF-7, MDA-MB-231) and ovarian (NCI-ADR) cancer cell lines.

Comp.	IC_50_ (µM) ^a^		
	MCF-7	MDA-MB-231	NCI-ADR
**HI 5**	1.15 ± 0.04	10.54 ± 0.43	9.17
Doxorubicin	6.81 ± 0.22	10.29 ± 0.72	ND ^b^

^a^ IC_50_ values are the mean ± S.D. of three separate experiments, ^b^ Not determined.

**Table 4:** Effect of **HI 5** on the expression levels of Bcl-2, Bax, active caspase-3, and p53 in MCF-7 cancer cells.

Compound	Bax (pg/mg of Total Protein)	Bcl-2 (ng/mg of Total Protein)	Bax/Bcl-2	Caspase-3 (pg/mg)	p53 (pg/mg)
**HI 5**	297.4 ± 11.1	2.56 ± 0.20	116.2 ± 0.8	387.5 ± 13.7	673.2 ± 41.0
Control	38.3 ± 2.2	4.65 ± 0.23	8.2 ± 0.7	35.92 ± 1.8	41.26 ± 2.7

**Table 5:** Results of cell cycle analysis in MCF-7 expressed by (%) of cell in each phase when treated with compound **HI 5.**

Comp.	%G0-G1	%S	%G2-M	%Sub-G1
**HI 5**	41.54 ± 0.8	22.17 ± 1.0	36.29 ± 1.2	17.34 ± 0.2
Control	57.26 ± 0.2	28.59 ± 0.5	14.15 ± 1.0	1.79 ± 0.8

**Table 6:** **HI 5***syn*/*anti*-isomers and co-crystallized ligand energies (kJ/mol).

Compound	Van der Waals	Electrostatic	Polar Solvation	SASA	Binding Energy
Co-crystallized ligand ^1^	−178.546 ± 10.295	−20.344 ± 7.826	107.937 ± 11.584	−15.750 ± 0.720	−106.703 ± 3.411
**HI 5** (*syn*-isomer)	−186.753 ± 9.303	−53.024 ± 6.399	165.947 ± 8.579	−17.805 ± 0.788	−91.636 ± 12.345
**HI 5** (*anti*-isomer)	−102.060 ± 25.028	−28.865 ± 8.671	82.992 ± 56.836	−10.440 ± 2.166	−58.374 ± 47.381

^1^ 4-(5-Bromo-2-oxo-2*H*-indol-3-ylazo)-benzenesulfonamide.

## Data Availability

The data presented in this study are available on request from the corresponding author.

## Supplementary Materials

The following are available online, Figure S1: ^1^H-NMR chart for compound **HI 5**, Figure S2: ^1^H-NMR expanded chart for compound **HI 5**, Figure S3: ^13^C-NMR chart for compound **HI 5**.

## Sample Availability

Sample of the compound **HI 5** is available from the authors.
