# Supplementary material for: A New CDK2 Inhibitor with 3-Hydrazonoindolin-2-One Scaffold Endowed with Anti-Breast Cancer Activity: Design, Synthesis, Biological Evaluation, and In Silico Insights
Source: Molecules. 2021 Jan 14;26(2):412. doi: 10.3390/molecules26020412 (PMC7830330; doi:10.3390/molecules26020412)
Supplement: Supplementary file 1 [file molecules-26-00412-s001.pdf]

S1: 1HNMR chart for compound HI 5.

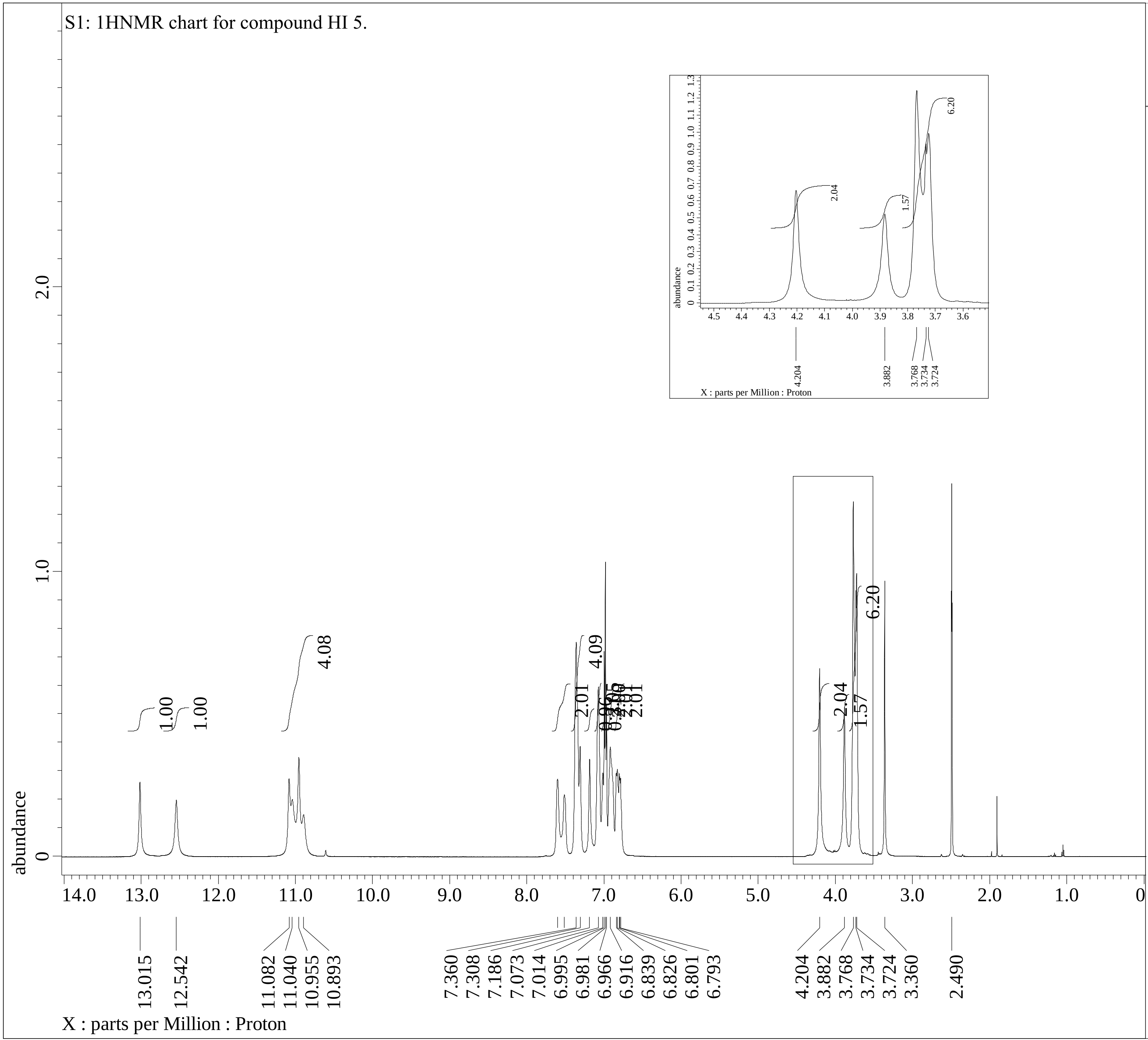

Mansoura University
JEOL
NMR Unit
ECA- 500 II

```
Filename          = Dr.Al-Sanea Mohammed_HI
Author            = proton.jxp
Experiment        = Dr.Wagdy Mohammed_3H_5_O
Sample_Id        = DMSO-D6
Solvent           = 23-SEP-2019 11:17:35
Creation_Time     = 17-OCT-2019 14:23:19
Revision_Time     = 17-OCT-2019 14:23:26
Current_Time

Comment           = single_pulse
Data_Format       = 1D COMPLEX
Dim_Size          = 13107
Dim_Title         = Proton
Dim_Units         = [ppm]
Dimensions        = X
Site              = JNM-ECA500II
Spectrometer      = DELTA2_NMR

Field_Strength    = 11.7473579[T] (500[MHz])
X_Acq_Duration    = 1.4548992[s]
X_Domain          = 1H
X_Freq            = 500.15991521[MHz]
X_Offset          = 6[ppm]
X_Points          = 16384
X_Prescans        = 1
X_Resolution      = 0.68733284[Hz]
X_Sweep           = 11.26126126[kHz]
X_Sweep_Clipped   = 9.00900901[kHz]
Irr_Domain        = Proton
Irr_Freq          = 500.15991521[MHz]
Irr_Offset        = 5.0[ppm]
Tri_Domain        = Proton
Tri_Freq          = 500.15991521[MHz]
Tri_Offset        = 5.0[ppm]
Clipped           = FALSE
Scans             = 40
Total_Scans       = 40

Relaxation_Delay  = 5[s]
Recvr_Gain        = 38
Temp_Get          = 21.1[dC]
X_90_Width        = 14.5[us]
X_Acq_Time        = 1.4548992[s]
X_Angle           = 45[deg]
X_Atn             = 4.9[dB]
X_Pulse           = 7.25[us]
Irr_Mode          = Off
Tri_Mode          = Off
Dante_Presat      = FALSE
Initial_Wait      = 1[s]
Repetition_Time   = 6.4548992[s]
```

S2 : 1H NMR HI 5

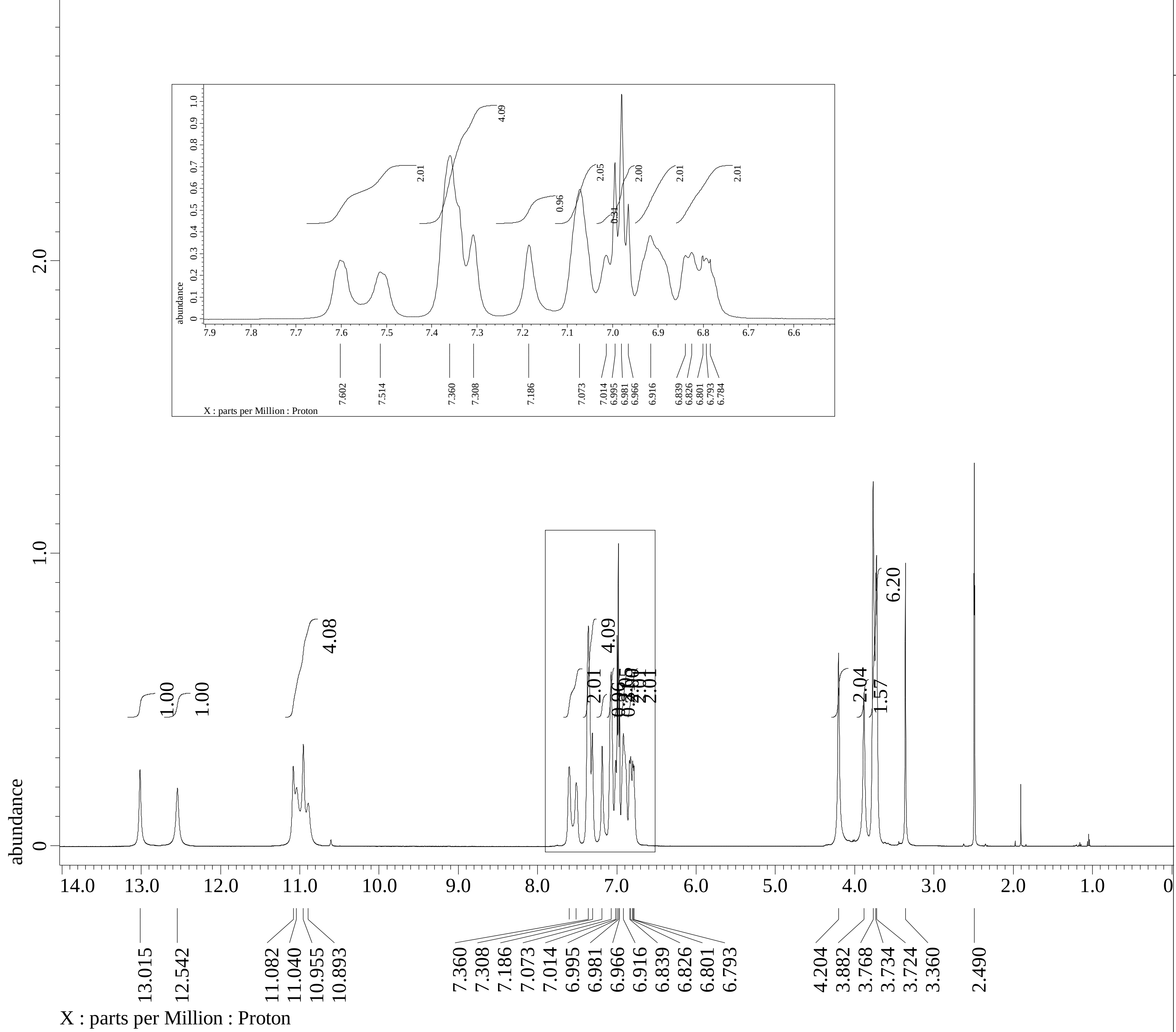

Mansoura University
JEOL ECA- 500 II
NMR Unit

Filename = Dr.Al-SANEA Mohammed_HI
Author = delta
Experiment = proton.jxp
Sample_Id = Dr.Al-SANEA
Solvent Mohammed_3H_5_OC
Creation_Time = DMSO-D6
Revision_Time = 23-SEP-2019 11:17:35
Current_Time = 17-OCT-2019 14:22:03
= 17-OCT-2019 14:22:40
Comment = single_pulse
Data_Format = 1D COMPLEX
Dim_Size = 13107
Dim_Title = Proton
Dim_Units = [ppm]
Dimensions = X
Site = JNM-ECA500II
Spectrometer = DELTA2_NMR

Field_Strength = 11.7473579[T] (500[MHz])
X_Acq_Duration = 1.4548992[s]
X_Domain = 1H
X_Freq = 500.15991521[MHz]
X_Offset = 6[ppm]
X_Points = 16384
X_Prescans = 1
X_Resolution = 0.68733284[Hz]
X_Sweep = 11.26126126[kHz]
X_Sweep_Clipped = 9.00900901[kHz]
Irr_Domain = Proton
Irr_Freq = 500.15991521[MHz]
Irr_Offset = 5.0[ppm]
Tri_Domain = Proton
Tri_Freq = 500.15991521[MHz]
Tri_Offset = 5.0[ppm]
Clipped = FALSE
Scans = 40
Total_Scans = 40

Relaxation_Delay = 5[s]
Recvr_Gain = 38
Temp_Get = 21.1[dC]
X_90_Width = 14.5[us]
X_Acq_Time = 1.4548992[s]
X_Angle = 45[deg]
X_Atn = 4.9[dB]
X_Pulse = 7.25[us]
Irr_Mode = Off
Tri_Mode = Off
Dante_Presat = FALSE
Initial_Wait = 1[s]
Repetition_Time = 6.4548992[s]

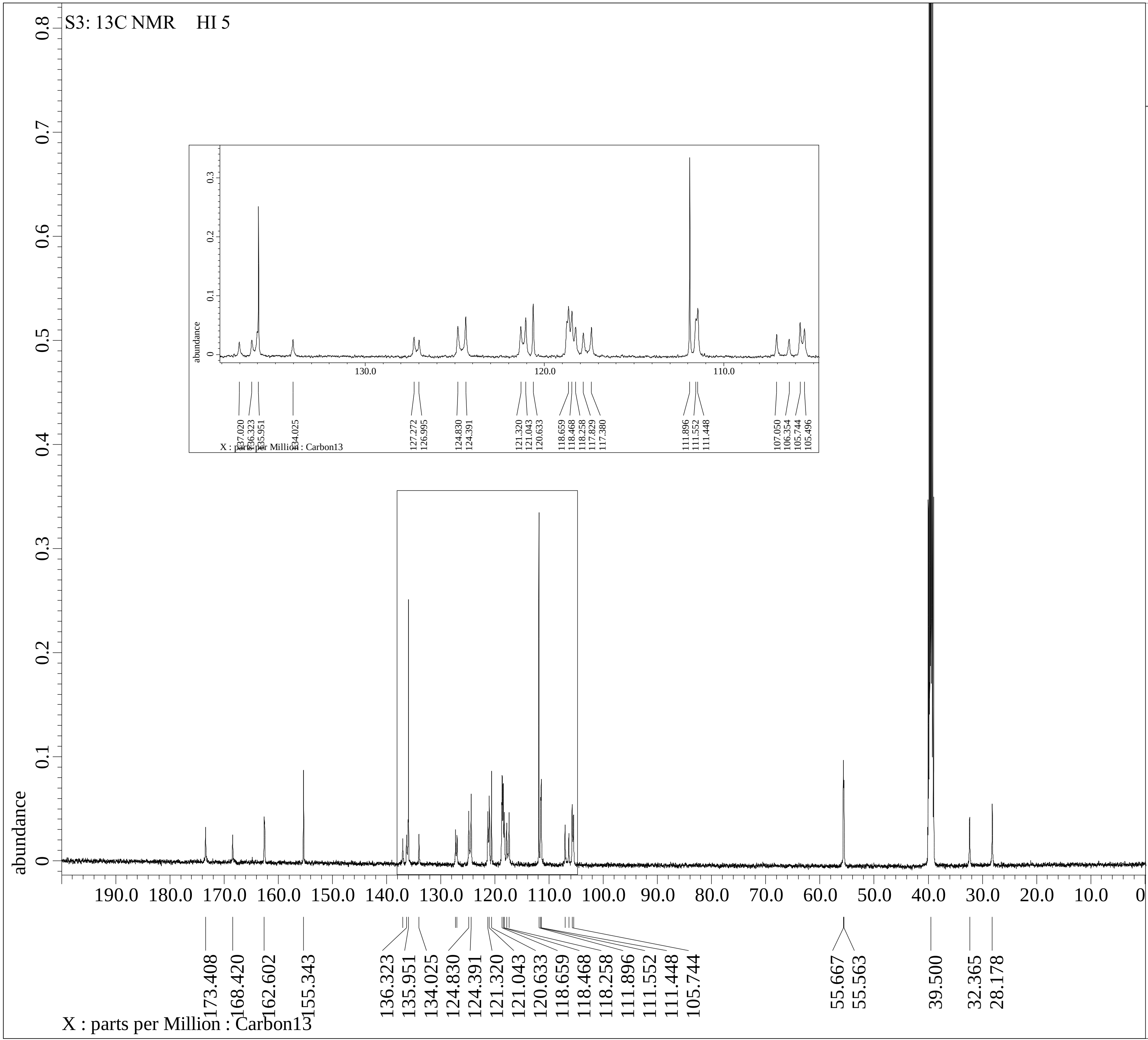

Mansoura University
JEOL NMR Unit
ECA- 500 II

```
Filename           = Dr. Al-SANEA
Author             = carbon.jxp
Experiment         = Dr.Al-SANEA = DMSO-D6
Sample_Id          = 23-SEP-2019 17:59:32
Solvent            = 24-SEP-2019 13:03:31
Creation_Time      = 24-SEP-2019 13:04:22
Revision_Time
Current_Time

Comment            = single pulse decoupled g
Data_Format        = 1D COMPLEX
Dim_Size           = 26214
Dim_Title          = Carbon13
Dim_Units          = [ppm]
Dimensions         = X
Site               = JNM-ECA500II
Spectrometer       = DELTA2_NMR

Field_Strength     = 11.7473579[T] (500[MHz])
X_Acq_Duration     = 0.83361792[s]
X_Domain           = 13C
X_Freq             = 125.76529768[MHz]
X_Offset           = 100[ppm]
X_Points           = 32768
X_Prescans         = 4
X_Resolution       = 1.19959034[Hz]
X_Sweep            = 39.3081761[kHz]
X_Sweep_Clipped    = 31.44654088[kHz]
Irr_Domain         = Proton
Irr_Freq           = 500.15991521[MHz]
Irr_Offset         = 5.0[ppm]
Clipped            = FALSE
Scans              = 3700
Total_Scans        = 3700

Relaxation_Delay   = 2[s]
Recvr_Gain         = 56
Temp_Get           = 18.9[dC]
X_90_Width         = 52.78[us]
X_Acq_Time         = 0.83361792[s]
X_Angle            = 30[deg]
X_Atn              = 9.7[dB]
X_Pulse            = 17.59333333[us]
Irr_Atn_Dec        = 20.948[dB]
Irr_Atn_Noe        = 20.948[dB]
Irr_Noise          = WALTZ
Irr_Pwidth         = 92[us]
Decoupling         = TRUE
Initial_Wait       = 1[s]
Noe                = TRUE
Noe_Time           = 2[s]
Repetition_Time    = 2.83361792[s]
```
